# Drug Design Studio (DDS) 2.0: A Unified Platform for Network Pharmacology Integrated with Docking and Virtual Screening Workflow for Covalent/Non-Covalent Binders

**DOI:** 10.3390/ijms27177874

**Published:** 2026-09-03

**Authors:** Mahmoud E. Soliman

**Affiliations:** Molecular Bio-Computation and Drug Design Laboratory, School of Health Sciences, University of KwaZulu-Natal, Westville Campus, Durban 4000, South Africa; soliman@ukzn.ac.za; Tel.: +27-(0)-312607413

**Keywords:** network pharmacology, drug discovery, protein–protein interaction network, hub genes, molecular docking, virtual screening, covalent inhibitors, integrated software platform

## Abstract

Network pharmacology has become a central paradigm in modern drug discovery, replacing the reductionist “one drug, one target” view with a systems-level understanding of how compounds engage networks of proteins that are linked to disease. Despite its impact, a typical network-pharmacology study remains fragmented and technically demanding: researchers must query several independent databases, install and reconcile multiple standalone tools for target collection, network construction, hub-gene ranking and pathway enrichment, and then manually bridge the results into structure-based follow-up such as molecular docking. This fragmentation is a persistent barrier, particularly for experimental and non-specialist users. Here, we present the network-pharmacology module of Drug Design Studio (DDS) 2.0, a unified, user-friendly platform that streamlines the entire workflow—disease target retrieval, compound–target prediction, shared-target identification, protein–protein interaction (PPI) network construction, hub-gene ranking and Gene Ontology/pathway enrichment—within a single guided interface, consolidating steps that otherwise require several separate tools. Crucially, DDS 2.0 links the resulting hub genes directly to the docking and virtual-screening engine introduced in the previous DDS releases: representative experimental structures and mutant forms—for instance, resistance-conferring variants found in drug-resistant strains—of the target proteins are selected and streamed into a docking-ready workspace, with dedicated support for covalent binders. We validate the module against four independent published network-pharmacology studies spanning diverse diseases; DDS reproduces the reported hub genes with a mean recovery (recall) of 0.85 (range 0.80–0.90) and a mean Jaccard index of 0.74, and recovers the corresponding target sets and enriched pathways. DDS 2.0 thus delivers an integrated route from systems-level analysis to structure-based drug design. DDS 2.0 is freely and publicly accessible. Comprehensive user documentation is built directly into DDS and can be accessed at any time from the Documentation panel.

## 1. Introduction

Drug discovery has historically been guided by the “one drug, one target, one disease” model. Although this reductionist framework has produced many successful therapeutics, it fails to capture the polypharmacological reality that most bioactive small molecules engage multiple proteins, and that complex diseases arise from the perturbation of interconnected molecular networks rather than single genes [1,2]. In recent years, computational approaches to drug discovery have advanced rapidly and become increasingly interconnected. Network and systems pharmacology have matured from descriptive tools into frameworks for uncovering the causal, multi-target mechanisms that underlie complex diseases [1,3,4,5], while parallel advances in artificial intelligence-assisted methods have strengthened target identification, virtual screening and lead optimisation across the discovery pipeline [6,7]. Collectively, these developments have shifted the field away from single-target, single-tool analyses and toward integrated, multi-target and systems-level strategies—a shift that motivates unified platforms capable of combining these complementary approaches within a single, reproducible workflow.

Despite its conceptual power, executing a network-pharmacology study in practice is fragmented, laborious and error-prone [8,9]. These failures are concrete: manual data transfer between packages, file-format incompatibilities, inconsistent parameters across tools, database-version mismatches, and the user-dependent mistakes that accumulate across a long multi-step analysis—each a documented source of irreproducibility that an integrated pipeline is positioned to remove. A standard workflow requires the researcher to (i) collect disease-associated genes from resources such as Open Targets [10], DisGeNET [11], GeneCards or the Comparative Toxicogenomics Database (CTD) [12]; (ii) collect or predict compound targets from databases such as the Drug–Gene Interaction Database (DGIdb) [13], ChEMBL [14], SwissTargetPrediction [15] or TCMSP [16]; (iii) reconcile gene identifiers across sources, which frequently disagree on symbols and synonyms; (iv) compute the intersection of compound and disease targets; (v) build a protein–protein interaction (PPI) network, typically by exporting the shared genes to STRING [17] and importing the result into Cytoscape 3.10.4 [18]; (vi) rank hub genes using a centrality plug-in such as cytoHubba [19]; and (vii) perform Gene Ontology (GO) [20] and KEGG pathway [21] enrichment through a separate tool such as Enrichr [22], DAVID or clusterProfiler. Each of these steps lives in a different web service, desktop application or scripting environment, each with its own input format, identifier conventions and output layout. The user must install, learn and maintain several packages, manually transfer intermediate files between them, and stitch the results together by hand. This tooling burden slows research, hampers reproducibility, and places a particularly high barrier in front of experimental biologists and medicinal chemists who are not computational specialists.

A second, deeper gap follows the analysis itself. Network pharmacology identifies which proteins matter, but it does not, on its own, take the researcher to the next logical step—structure-based investigation of how the compound engages those proteins. Bridging hub genes to molecular docking requires yet another manual chain: mapping each gene to a protein, searching the Protein Data Bank (PDB) [23], judging which of the many available structures is the most suitable representative (a decision that usually demands a literature search), downloading and preparing it, and configuring a docking box. This hand-off is tedious and is rarely streamlined, so the systems-level and structure-based halves of a drug-discovery campaign remain disconnected in most workflows.

The objectives of this work are threefold: (i) to develop an integrated, guided software platform—the network-pharmacology module of DDS 2.0—that integrates the complete workflow from in-application compound–disease target construction, through protein–protein interaction network analysis and hub-gene prioritisation, mutation-aware experimental-structure selection and covalent-aware molecular docking and virtual screening; (ii) to benchmark this network-pharmacology pipeline quantitatively against independent published studies; and (iii) to make the entire workflow accessible and reproducible within a single application.

Drug Design Studio (DDS) was introduced as a cross-platform desktop (Microsoft Windows and Apple macOS) application that unifies structure-based drug design—receptor preparation, molecular docking, covalent and non-covalent virtual screening, and protein–ligand interaction analysis—behind a single guided interface, removing the need to assemble separate command-line tools for a docking campaign. Building on that foundation, we here present a substantial extension: a complete network-pharmacology module in DDS 2.0. The module streamlines the full network-pharmacology protocol—from disease and compound–target assembly, through PPI network construction and hub-gene ranking, to GO/KEGG enrichment—inside the same application. More importantly, the module is not a standalone add-on: its hub-gene output is integrated directly with the docking and virtual-screening engine of the earlier DDS release. Representative experimental structures for the top hub genes—wild-type or, where relevant, a specific point-mutant or drug-resistant form—are selected and delivered, docking-ready, into the DDS docking workspace, together with its covalent-docking protocols, thereby closing the loop from network-level analysis to structure-based design.

To our knowledge, no single existing platform provides this complete, purpose-built pipeline—from in-application compound–disease target construction, through network analysis and hub-gene identification, to mutation-aware experimental-structure selection and covalent-aware molecular docking and virtual screening—within one guided environment.

## 2. Results and Discussion

### 2.1. Overview

**Figure 1:**
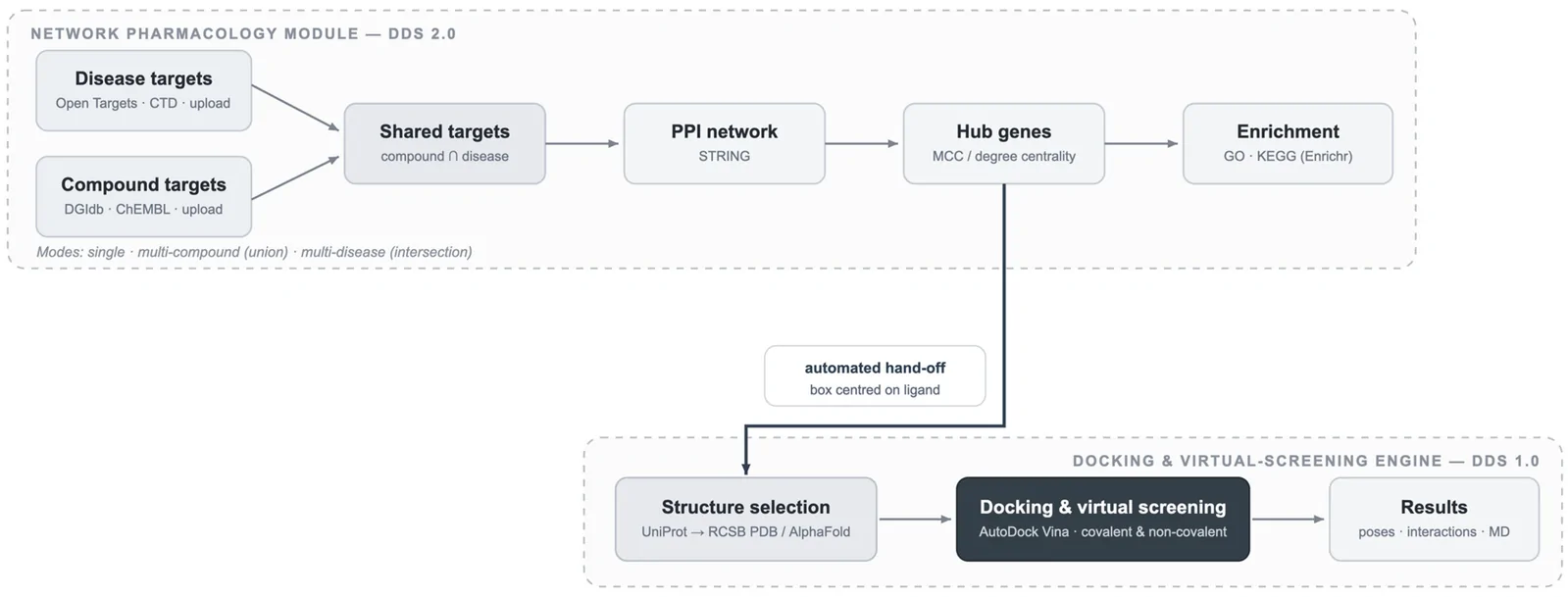
Overview of the Drug Design Studio (DDS) 2.0 network-pharmacology workflow and its integration with the docking and virtual-screening engine. Disease-associated genes (Open Targets, CTD, or user-uploaded lists) and predicted compound targets (DGIdb, ChEMBL, or user-uploaded lists) are assembled and intersected to give the shared targets, from which a STRING protein–protein interaction network is built; hub genes are ranked by centrality (MCC or degree), and the shared-target set is submitted for GO/KEGG functional enrichment. Single, multi-compound (union) and multi-disease (intersection) modes are supported. The top hub genes are resolved to a representative experimental structure (UniProt → RCSB PDB, with an AlphaFold fallback) and handed off, docking-ready, to the DDS docking and virtual-screening engine (AutoDock Vina; covalent and non-covalent), with the search box pre-centred on the co-crystallised ligand, producing docked poses, interaction analyses and molecular-dynamics-ready exports.

**Figure 2:**
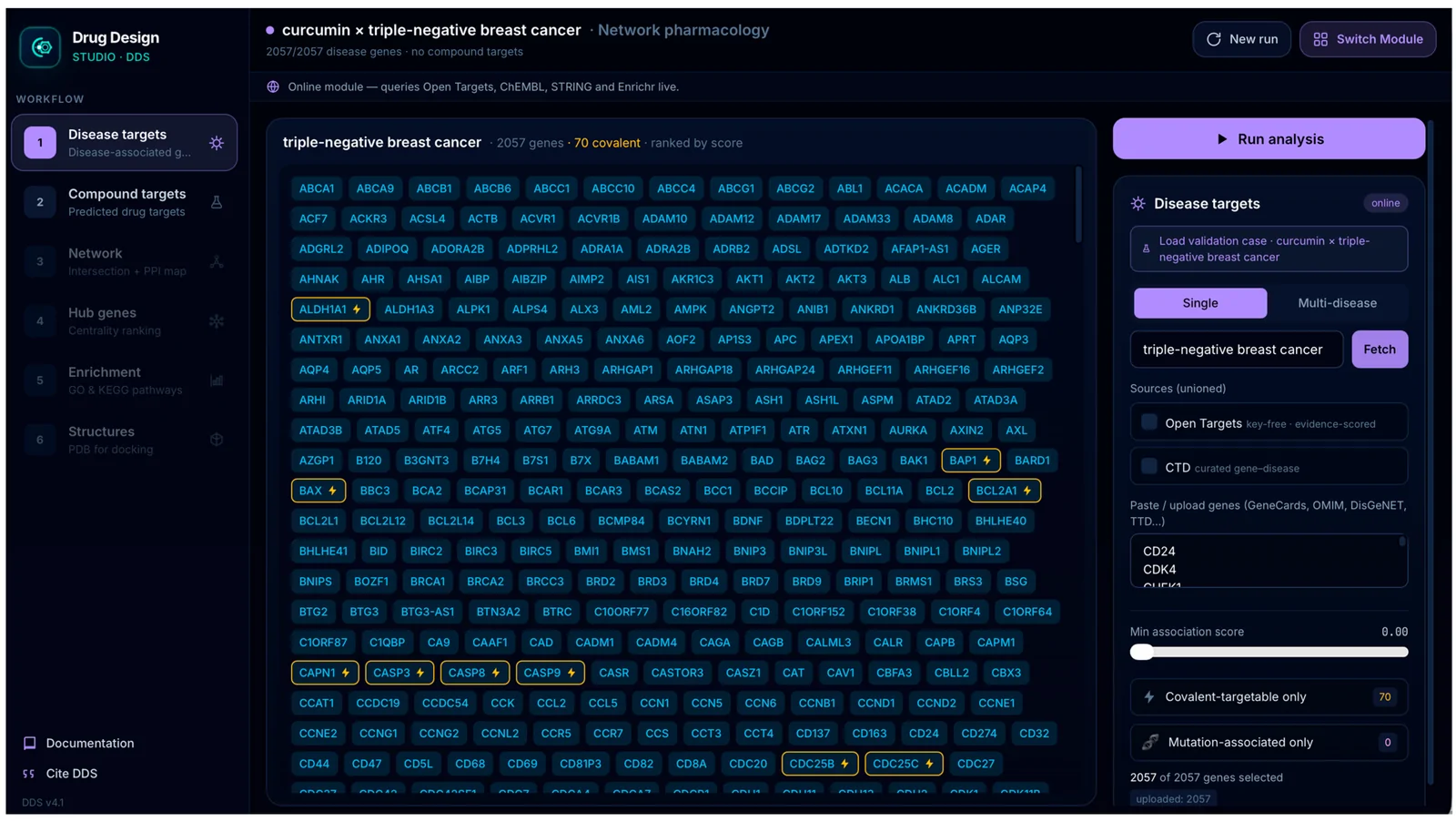
The Drug Design Studio (DDS) 2.0 network-pharmacology module interface, shown at the disease target step of the curcumin × triple-negative breast cancer benchmark. Left: the six-step guided workflow rail (disease targets → compound targets → network → hub genes → enrichment → structures). Centre: the disease-associated genes for the analysis—here the 2057 triple-negative breast carcinoma target genes of the reference study, loaded into DDS via the one-click validation case, with the 70 covalent-targetable proteins. Right: the controls for the step, including the one-click validation-case loader, a Single/Multi-disease toggle, a disease-name entry with a Fetch action, the selectable source databases (Open Targets, CTD), and a paste/upload field for target lists from external resources (GeneCards, OMIM, DisGeNET, TTD)—used here to supply the reference study’s target set with the online source databases switched off—together with a minimum-association-score cut-off, a covalent-targetable-only filter (70 proteins) and a mutation-associated-only filter. The top bar provides the new run and switch module controls, while the run analysis control heads the right-hand panel; the banner indicates that the module can query the public databases (Open Targets, ChEMBL, STRING, Enrichr) live.

The DDS 2.0 network-pharmacology module implements the complete network-pharmacology protocol described above within a single guided application and connects it directly to the DDS docking and virtual-screening engine. In practice, a study that would conventionally require the user to visit and reconcile Open Targets, DGIdb, ChEMBL, STRING, cytoHubba, Enrichr, the PDB and a docking package—installing and learning several tools and manually transferring intermediate files—is completed inside one interface, with every intermediate and final result available for interactive inspection and one-click export. The overall workflow and the module interface are shown in Figure 1 and Figure 2. The figures presented here are screenshots of the actual curcumin–triple-negative breast cancer (TNBC) benchmark run in DDS 2.0 and therefore correspond directly to the reported results [24]. The underlying data and the generated network-pharmacology report are provided as Supplementary Material File S1 (data sheets) and File S2 (the DDS-generated report), respectively. In addition, the complete benchmarking of DDS 2.0 against four independent published network-pharmacology studies—with the full target lists, hub-gene comparisons and quantitative validation metrics (hub-gene recall, Jaccard index and hub-rank correlation)—is provided in Supplementary Material File S3.

### 2.2. Benchmark Case Study: Curcumin in Triple-Negative Breast Cancer

**Figure 3:**
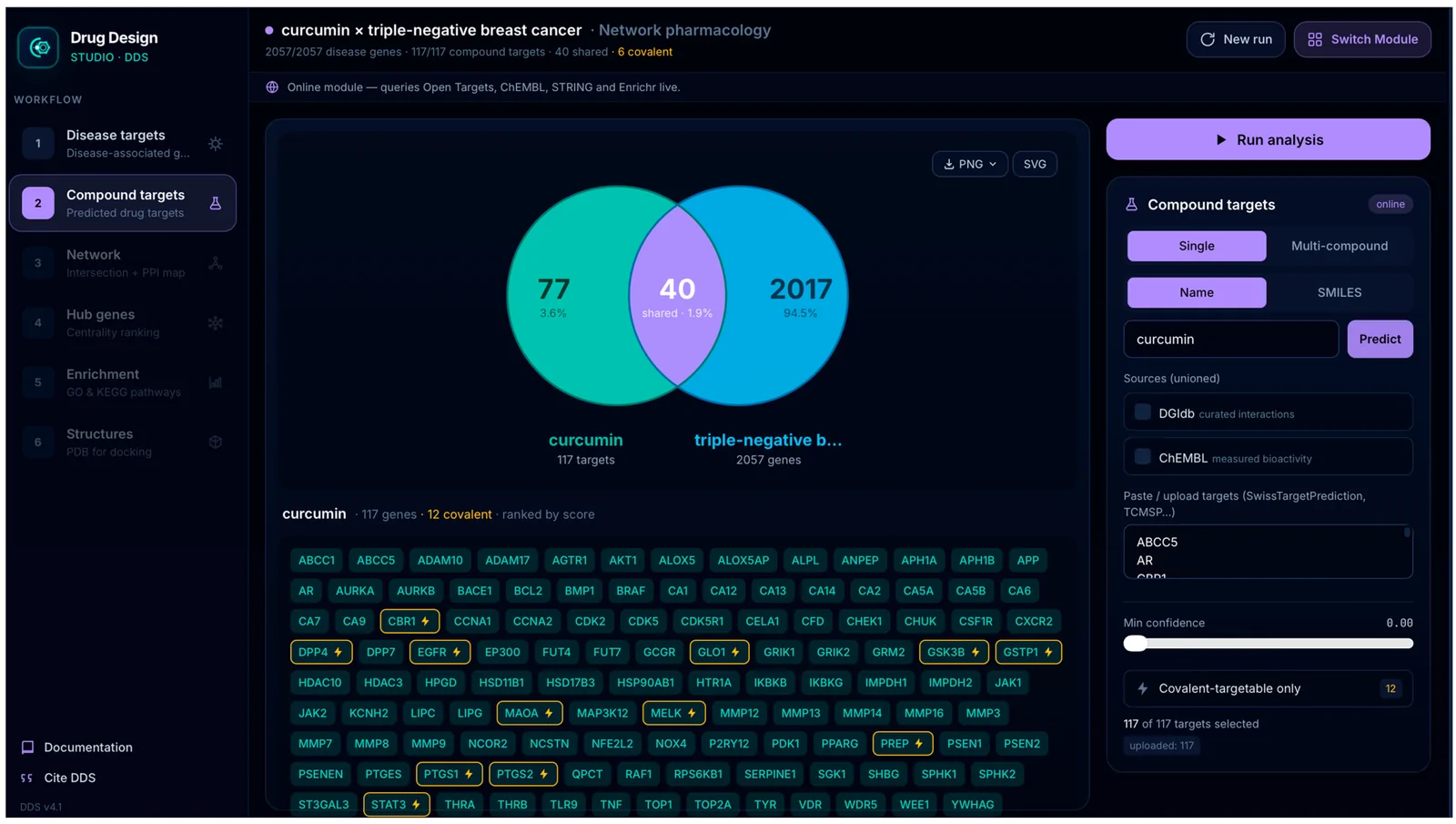
Compound–target prediction and shared-target identification in the DDS 2.0 network-pharmacology module (curcumin × triple-negative breast cancer). The central Venn diagram shows the intersection of the curcumin target set with the triple-negative breast carcinoma disease-gene set: of the 117 curcumin targets and 2057 disease genes, 77 are unique to curcumin and 2017 to the disease, with 40 shared targets (compound ∩ disease; 1.9% overlap)—the candidate therapeutic targets carried forward into the network analysis—of which 6 are covalent-targetable. Below the diagram, the curcumin target list is shown, with 12 of the 117 curcumin targets flagged as covalent-targetable proteins. Right: the compound–target controls—a Single/Multi-compound toggle, a Name/SMILES input mode with a Predict action, the selectable source databases (DGIdb curated interactions; ChEMBL measured bioactivity; here left unchecked), a paste/upload field used to supply the reference study’s curcumin target set (SwissTargetPrediction, TCMSP), a minimum-confidence cut-off, and a covalent-targetable-only filter (12 proteins). The Venn diagram is exportable as PNG (transparent/white/dark) or vector SVG.

As a benchmark, DDS 2.0 was validated against four independent published network-pharmacology systems. The curcumin–triple-negative breast cancer (TNBC) analysis) [24],—a well-studied system for the multi-target action of a natural product—is reproduced in detail here as a worked example; the remaining three systems (berberine in ischaemic stroke, baicalein in peritoneal fibrosis and quercetin in gastric cancer) are detailed, with full target lists, hub-gene comparisons and overlap metrics, in Supplementary Material File S3. Herein, to ensure a fully consistent comparison, the reference study’s own target sets were supplied directly to DDS 2.0: the 117 curcumin targets and 2057 triple-negative breast cancer (TNBC) targets reported in that study were loaded through the module’s target-upload fields, with the external source databases switched off, and the same PPI resource (STRING) was used. This isolates the DDS analytical pipeline—target intersection, STRING network construction, hub-gene ranking and functional enrichment—and evaluates it on identical inputs against the published results, removing any confound arising from differing target-prediction databases. The analysis was run end-to-end in DDS and is provided in the module as a one-click validation case: the PPI network was built with STRING at a confidence of 0.4, hub genes were ranked by Maximal Clique Centrality (MCC, cytoHubba) to match the reference study, and GO/KEGG enrichment was computed with Enrichr. DDS intersected the 117 curcumin targets with the 2057 TNBC targets to yield 40 shared candidate therapeutic targets. The summary counts are given in Table 1, and the intersection is shown as a Venn diagram in Figure 3.

**Figure 4:**
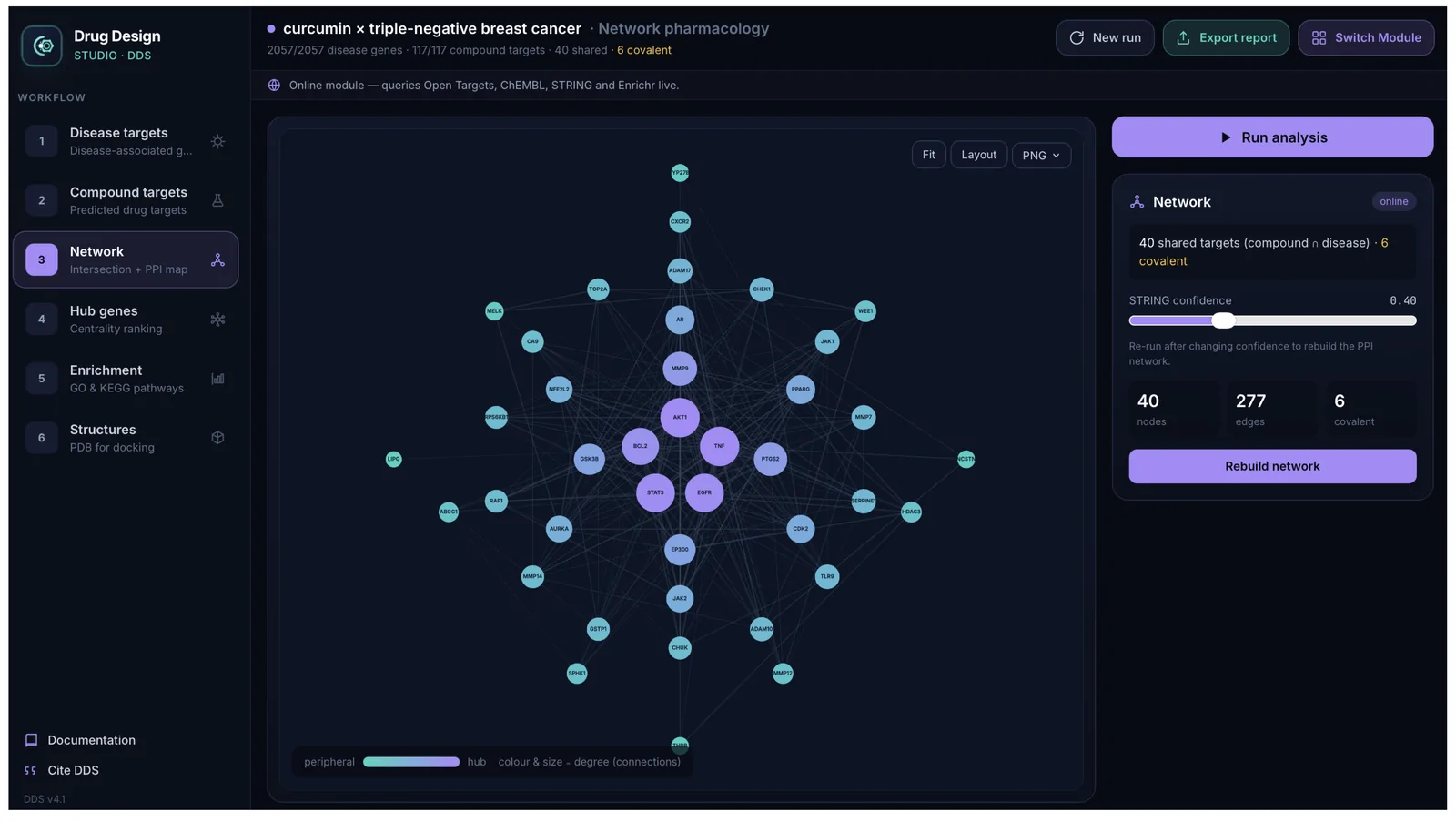
Protein–protein interaction (PPI) network of the shared targets in the DDS 2.0 network-pharmacology module (curcumin × triple-negative breast cancer). The 40 shared targets (compound ∩ disease) were submitted to STRING (confidence ≥ 0.40), producing a network of 40 nodes and 277 edges, of which 6 are covalent-targetable. Nodes are coloured and sized by degree (number of connections), from peripheral (teal) to hub (purple), so that the most connected, central genes—here AKT1, STAT3, TNF, EGFR, BCL2 and other core mediators—stand out as the candidate hub genes carried into the ranking step. Right: the network controls—a STRING-confidence slider (set to 0.40) with a Rebuild-network action, and the node, edge and covalent-target counts (40/277/6). The network view is exportable as PNG, with Fit and layout controls for arrangement.

**Figure 5:**
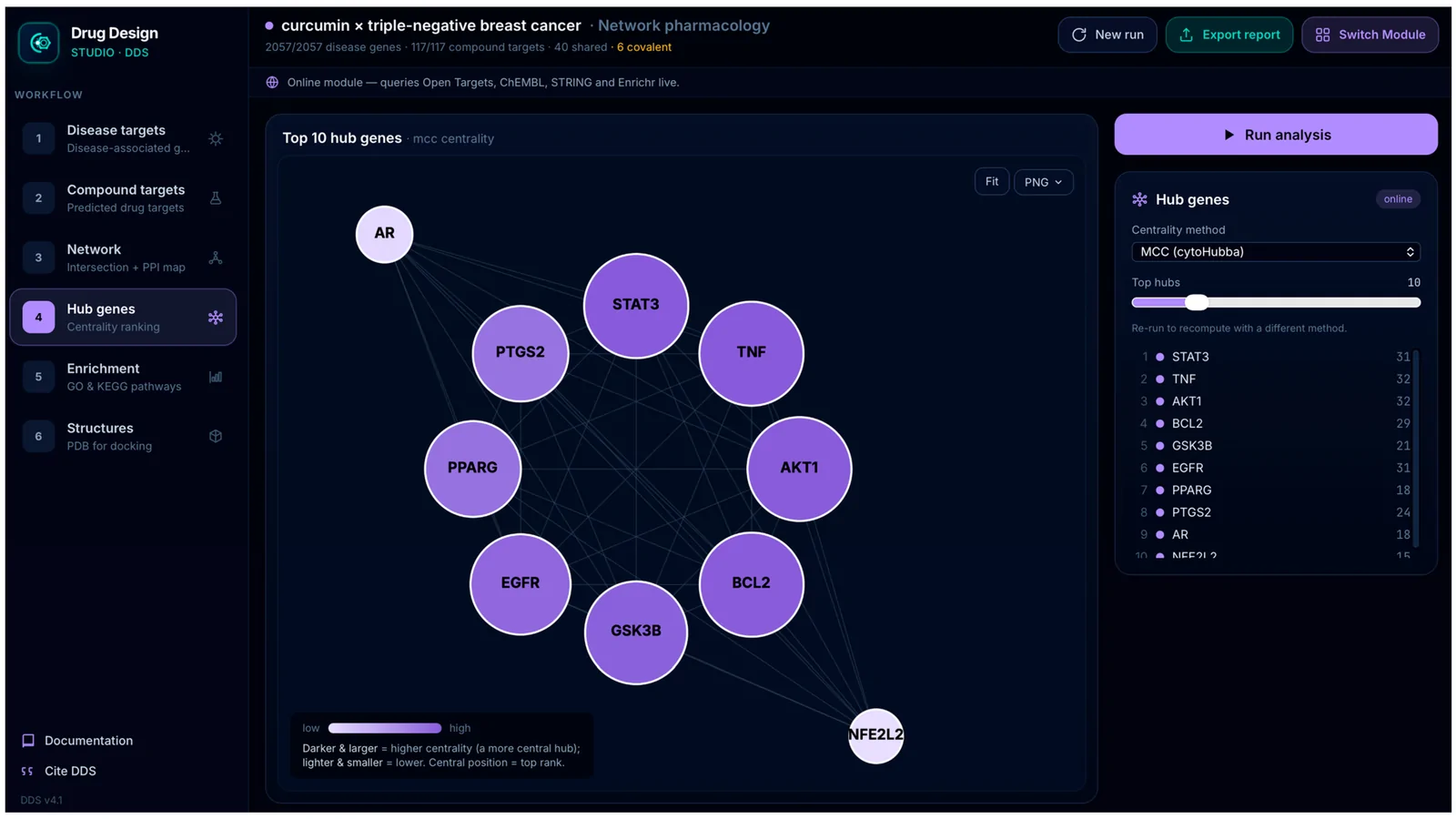
Top 10 hub genes ranked by Maximal Clique Centrality (MCC, cytoHubba) for the curcumin × triple-negative breast cancer case; degree, betweenness and closeness centrality are also user-selectable. Node darkness and size scale with centrality and central position denote higher rank, so the top-ranked hubs—STAT3, TNF, AKT1, BCL2, GSK3B, EGFR, PPARG, PTGS2, AR and NFE2L2—occupy the centre. These are the hub genes recovered by the benchmark reproduction; the full per-case validation metrics are reported in the Supplementary Material File S3.

PPI network and hub genes. The 40 shared targets were submitted to STRING and assembled into a PPI network of 40 nodes and 277 edges. Topological ranking identified a set of highly connected hub genes dominated by well-established TNBC and curcumin-responsive signalling regulators. The top hub genes, ranked by Maximal Clique Centrality (MCC, cytoHubba) to match the reference study, are listed in Table 2; the PPI network and the cytoHubba-style hub-gene figure are shown in Figure 4 and Figure 5.

Ranked by Maximal Clique Centrality (MCC, cytoHubba)—the same method used in the reference study—the recovered hubs (STAT3, AKT1, TNF, PTGS2, EGFR, PPARG, NFE2L2 and GSK3B) are consistent with the central mediators reported for curcumin activity in TNBC in the reference study [24] and in the wider literature, confirming that DDS recovers the biologically meaningful core of the network. Directly comparing the two studies, eight of the ten hub genes reported by the reference study (STAT3, AKT1, TNF, PTGS2, EGFR, PPARG, NFE2L2 and GSK3B) are recovered within the DDS top ten (recall = 0.80); the two remaining reference hubs (MMP9 and EP300) are also present in the DDS shared-target network, ranking just outside the top ten. Both are, nonetheless, well-established curcumin-associated targets in triple-negative breast cancer: MMP9 (matrix metalloproteinase-9) is a key mediator of extracellular-matrix degradation, tumour invasion and metastasis and a recognised downstream target of curcumin, whereas EP300 (the p300 histone acetyltransferase) is a transcriptional co-activator that curcumin is known to inhibit. Their position immediately below the MCC hub cut-off therefore reflects the topology of this specific high-confidence sub-network rather than biological insignificance, and both would be recovered at a marginally larger hub set. The two additional DDS hubs (BCL2 and AR) are likewise well-documented regulators of TNBC. DDS additionally offers degree, betweenness and closeness centrality as user-selectable alternatives.

**Figure 6:**
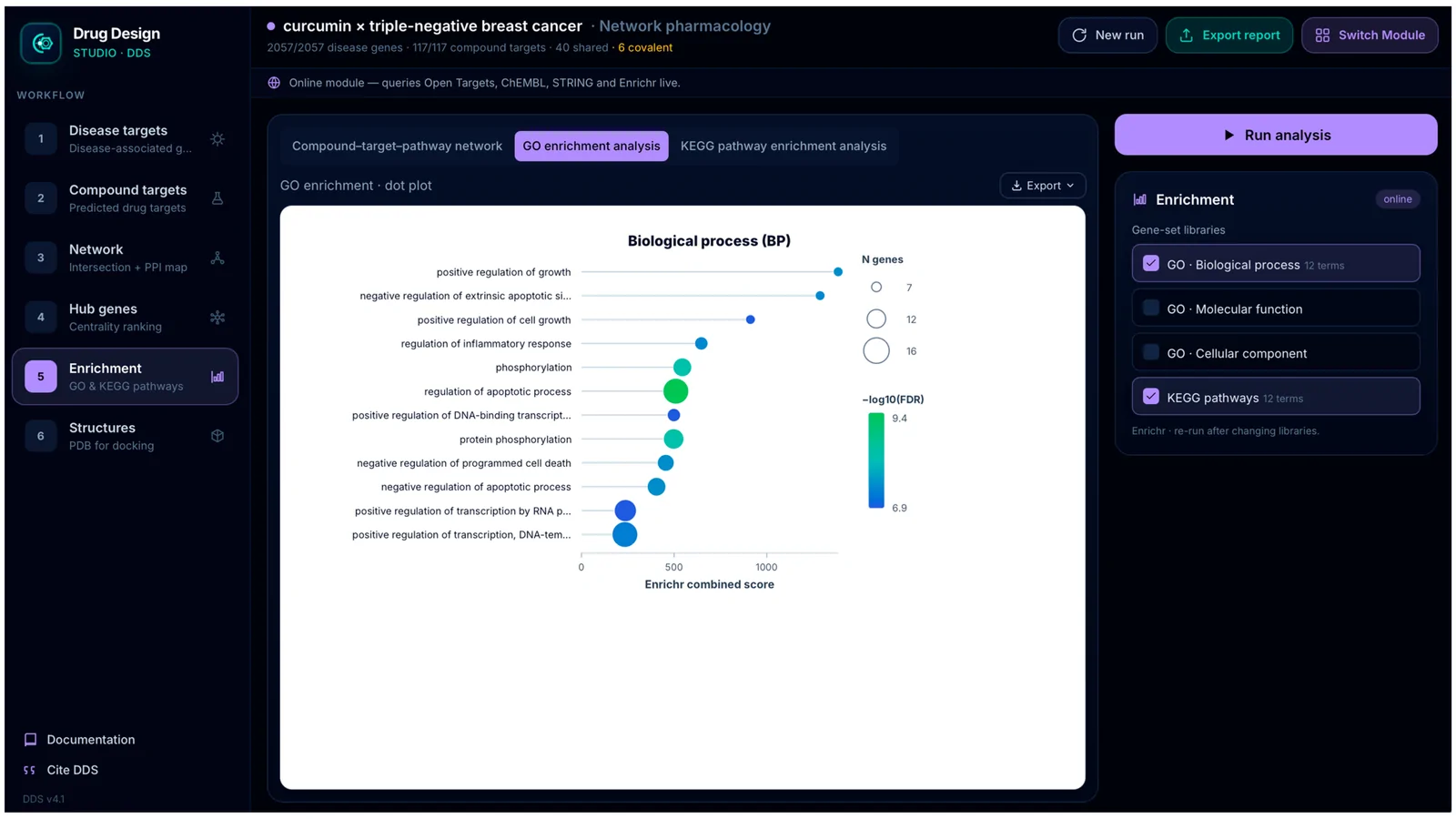
Gene Ontology (Biological Process) enrichment of the curcumin × triple-negative breast cancer shared-target set, shown as a dot plot in the DDS 2.0 module. GO-BP terms are ranked by Enrichr combined score (x-axis); dot size is proportional to the number of member genes and dot colour encodes −log10(FDR) (blue → green, per the legend). The enriched processes are dominated by the regulation of cell growth and proliferation (positive regulation of growth and of cell growth), the control of apoptosis (regulation of apoptotic process, negative regulation of extrinsic apoptotic signalling, negative regulation of programmed cell death), the regulation of inflammatory response, protein phosphorylation, and the regulation of transcription (positive regulation of transcription by RNA-polymerase-II and of DNA-templated transcription)—Biological Process consistent with the established anti-cancer activity of curcumin in triple-negative breast cancer. The GO Molecular Function and Cellular Component libraries can be added from the right-hand panel, and the figure is exportable as high-resolution PNG or vector SVG.

**Figure 7:**
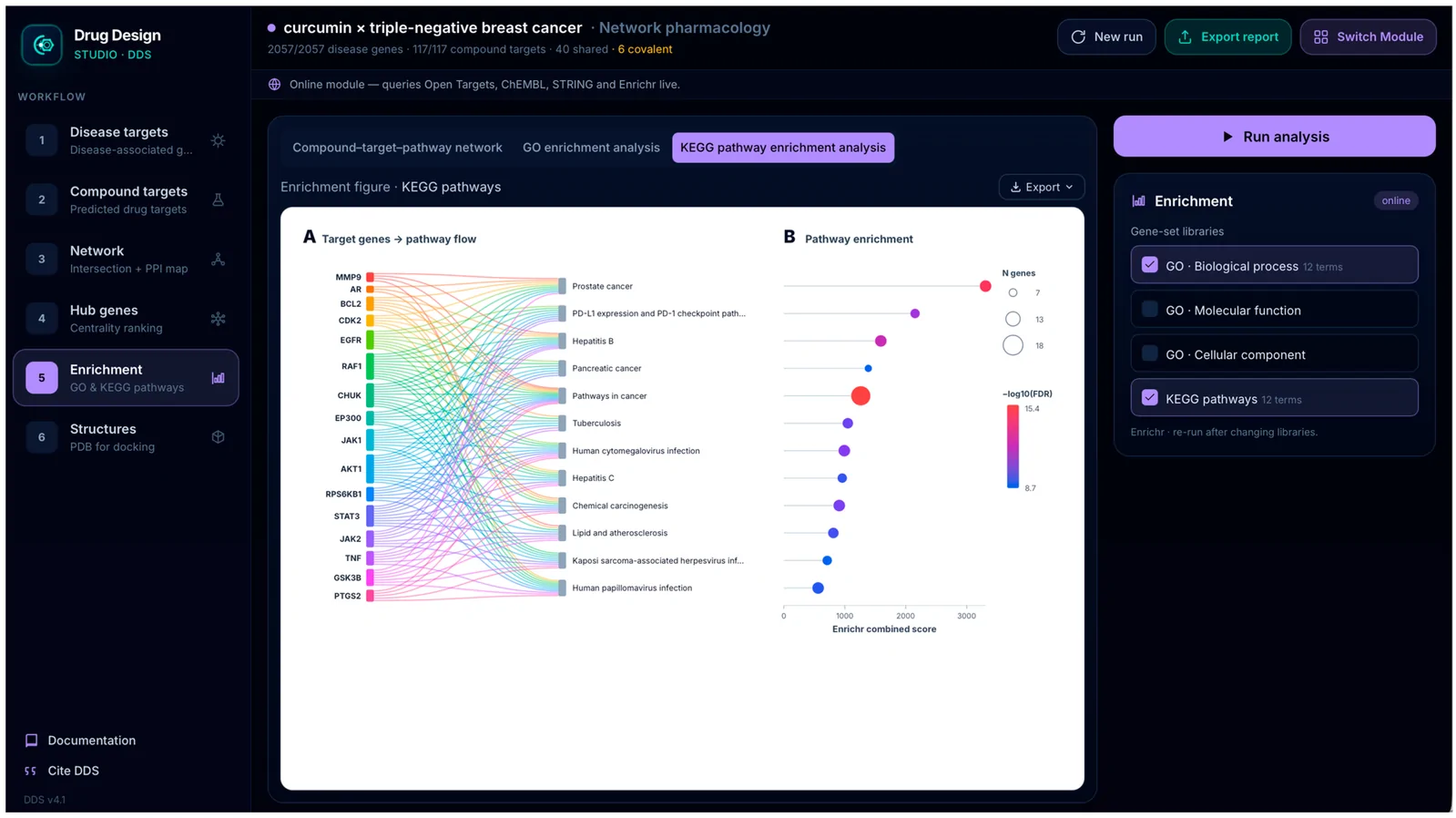
KEGG pathway enrichment of the curcumin × triple-negative breast cancer shared-target set in the DDS 2.0 module. (**A**) Sankey diagram of the flow from target genes (left; e.g., MMP9, AR, BCL2, EGFR, CHUK, AKT1, STAT3, TNF, PTGS2) to enriched KEGG pathways (right); coloured ribbons trace genes that contribute to multiple pathways. (**B**) Pathway-enrichment bubble plot: pathways ranked by Enrichr combined score (x-axis), with dot size proportional to the number of member genes and dot colour to −log10(FDR). The most strongly enriched terms are dominated by cancer pathways (pathways in cancer, Prostate cancer, Pancreatic cancer, Chemical carcinogenesis), immune-checkpoint signalling (PD-L1 expression and PD-1 checkpoint pathway in cancer), and cancer-associated viral infection pathways (Hepatitis B and C, human cytomegalovirus, Kaposi-sarcoma-associated herpesvirus and human papillomavirus infection), consistent with the anti-cancer and immunomodulatory pharmacology of curcumin in triple-negative breast cancer. The figure is exportable as high-resolution PNG or vector SVG.

Functional enrichment, GO and KEGG, of the shared-target set returned terms consistent with the known pharmacology of curcumin in cancer, including the regulation of transcription, programmed cell death and response to oxidative stress at the GO level, and cancer-associated signalling pathways at the KEGG level. Representative enriched terms are summarised in Table 3, and the enrichment figures (GO dot plot and KEGG hub-to-pathway Sankey/bubble figure) are shown in Figure 6 and Figure 7. Complete enrichment tables, with *p*-values, combined scores and member genes for every term and every library, are provided in the Supplementary Material File S3.

***Reproducibility assessment***: To benchmark the DDS pipeline against a published study, the reference study’s own disease- and compound–target sets were supplied to DDS through its data-upload interface and processed with the identical DDS network-pharmacology workflow. The resulting shared-target set, top hub genes and enriched GO/KEGG terms all agree closely with the published reference study [24]. A detailed side-by-side comparison of the key parameters is given in Table 4. The disease- and compound–target counts are essentially identical (2057 vs. 2060 and 117 vs. 118, respectively), and DDS reconstructs the same 40-gene compound ∩ disease intersection (40 vs. 40). Eight of the reference study’s ten hub genes are recovered within the DDS top ten (recall = 0.80), together with the same principal cancer pathways (pathways in cancer, and related cancer-type-specific and immune-checkpoint pathways). Beyond this worked example, the DDS pipeline was benchmarked against four independent published network-pharmacology studies spanning distinct disease areas (curcumin–triple-negative breast cancer, berberine–ischaemic stroke, baicalein–peritoneal fibrosis and quercetin–gastric cancer): across the four studies, DDS reproduced the published hub genes with a mean recovery (recall) of 0.85 (range 0.80–0.90) and a mean Jaccard index of 0.74, and the recovered hubs were significantly rank-correlated with the published ordering (Spearman ρ = 0.41, *p* = 0.03); the full per-study target lists, hub-gene comparisons and overlap metrics are provided in Supplementary Material File S3 (Tables S3.1 and S3.2, Figures S3.1 and S3.2). This concordance—both for the detailed curcumin–TNBC case and across the four independent benchmarks—demonstrates that consolidating the workflow into a single platform preserves the scientific results while eliminating the manual, multi-tool overhead.

**Figure 8:**
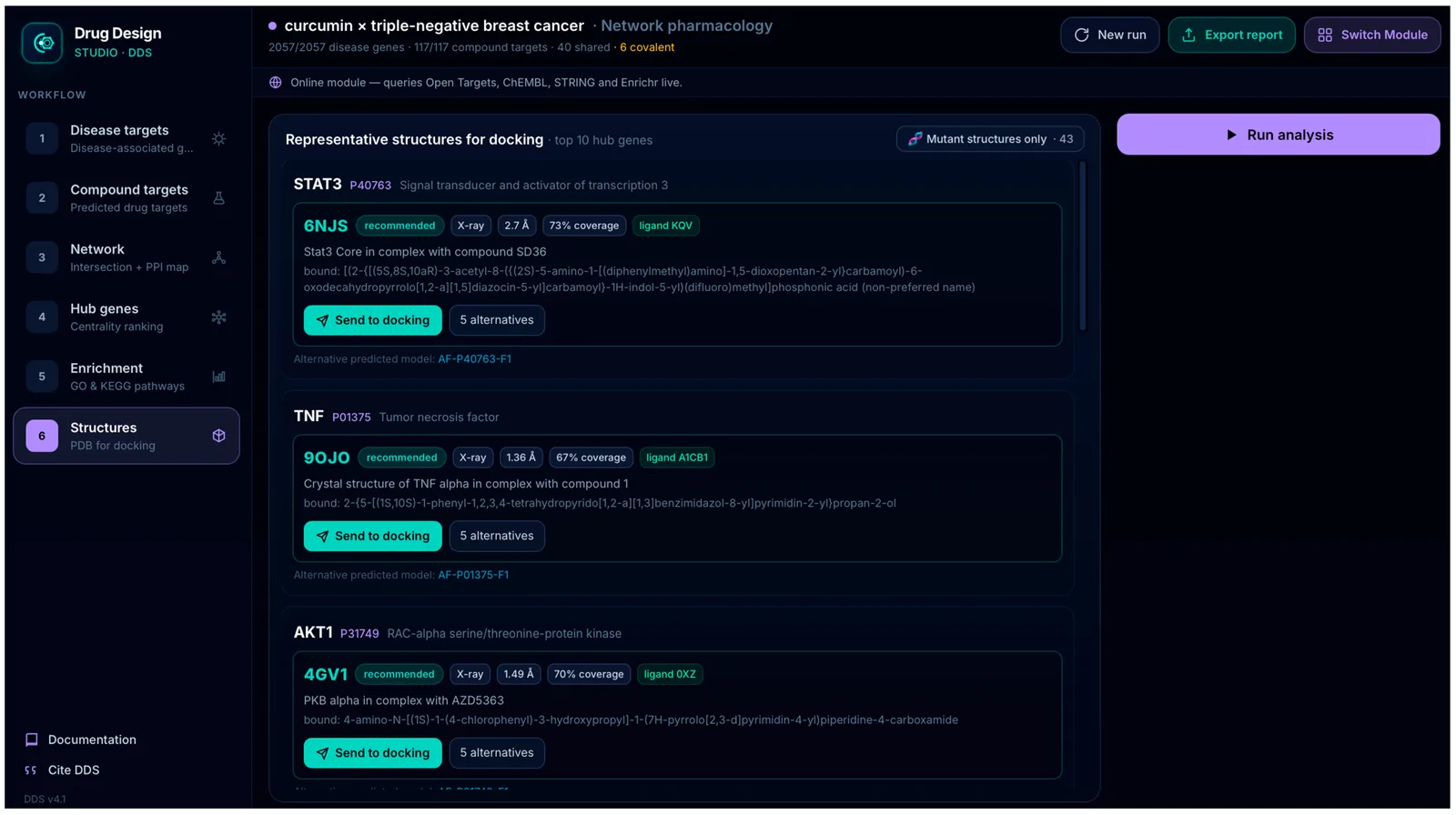
Representative-structure selection for docking in the DDS 2.0 Structures step, shown for the top 10 hub genes of the curcumin × triple-negative breast cancer network. For each hub gene, DDS resolves the gene to its reviewed human UniProt entry and ranks the available PDB structures for docking suitability by experimental method, resolution, domain coverage and bound ligand. Each card lists the recommended structure with its metadata badges (method, resolution, % domain coverage, bound ligand) and a short description, together with a one-click send to docking hand-off to the DDS docking module, a set of five ranked alternatives, and a link to the corresponding AlphaFold model shown beneath the card (e.g., AF-P40763-F1 for STAT3). A “Mutant structures only” filter—here surfacing 43 experimentally resolved mutant structures across the hub genes—additionally restricts the selection to mutant forms of the targets (Section 3.11). Representative examples: STAT3 → 6NJS (X-ray, 2.7 Å, 73% coverage, ligand KQV); TNF → 9OJO (X-ray, 1.36 Å, 67% coverage, ligand A1CB1); and AKT1 → 4GV1 (X-ray, 1.49 Å, 70% coverage, ligand 0XZ). For hub genes lacking a suitable experimental structure, DDS instead offers the corresponding AlphaFold model as the docking starting point, ensuring that even difficult targets have a defined structure.

Structure selection and docking hand-off: Finally, we exercised the network-pharmacology-to-docking integration on the benchmark hubs. For each top hub gene, DDS resolved a representative experimental structure and, where a suitable holo structure existed, pre-centred the docking box on the co-crystallised ligand; representative selections are given in Table 5. Selecting “send to docking” delivered the prepared receptor into the DDS docking workspace ready for docking of curcumin, with no manual PDB searching, preparation or box configuration required (Figure 8). For hub genes lacking a suitable experimental structure (e.g., largely disordered transcription factors), DDS instead offered the corresponding AlphaFold model, ensuring that even difficult targets have a defined starting point.

Taken together, the benchmark confirms that DDS 2.0 reproduces an established network-pharmacology result and carries that result forward into a docking-ready, covalent-aware structure-based workspace within the same application.

### 2.3. Comparison with Existing Approaches and Limitations

A defining feature of DDS, and one that distinguishes it from many contemporary computational drug-discovery platforms, is its transparency. As machine learning approaches have come to deliver much of the predictive performance in this field, a recognised difficulty is that the internal reasoning of such models is often hard to interrogate, which has motivated a substantial body of work on explainable artificial intelligence (XAI) in drug discovery [25]. It has been argued, however, that for high-stakes decisions the more robust route to trustworthy computation is not the post hoc explanation of opaque models but the use of methods that are inherently interpretable [26]. DDS follows this philosophy precisely: it is built upon established, deterministic and mechanistically grounded methods—compound–disease target intersection, protein–protein interaction network construction, topological hub-gene ranking, functional enrichment, and physics-based molecular docking—whose logic is transparent by construction and firmly rooted in the systems-level rationale of network pharmacology [2]. Each stage of the workflow yields a human-readable, inspectable result rather than an opaque score, and every prediction can be traced back to explicit biological and structural evidence and to the public databases from which it derives. In this way, the platform allows the user to appreciate not merely what is predicted, but the rationale by which the prediction is reached—a property that is essential for interpreting, validating and, ultimately, trusting the outcome of an in silico analysis.

Existing network-pharmacology practice relies on chaining several independent resources and tools, each requiring separate installation, identifier handling and manual data transfer, and stops short of structure-based follow-up. Some existing platforms address parts of this scope. Network-analysis environments—NetworkAnalyst [27], OmicsNet [28] and Cytoscape [18] (through apps such as cytoHubba, stringApp and ClueGO)—provide robust network construction, hub-gene ranking and enrichment, but operate on a user-supplied gene list and do not retrieve compound or disease targets, compute their intersection, or extend to structure selection, docking, virtual screening or covalent docking. General-purpose workflow engines such as KNIME and Galaxy can accommodate individual steps only through the manual assembly of separate extensions and external tools. DDS instead unifies the entire workflow within one guided application: it constructs the compound–disease target set in-app (Open Targets, CTD, ChEMBL and DGIdb, with cross-source gene-symbol reconciliation), supports multi-disease (shared-mechanism) and multi-compound (polypharmacology) analyses, ranks hub genes with a native implementation of the cytoHubba MCC measure, flags covalently targetable hubs, and streams the prioritised targets—with selected wild-type or mutant experimental structures—directly into covalent-aware docking and virtual screening. It is this integration across the full target-to-structure path, rather than any single constituent capability, that distinguishes the DDS platform.

Integration is not universally advantageous. By automating the transfer and format-handling steps between modules, DDS removes the mechanical and user-dependent errors of a fragmented pipeline while preserving the scientific decisions that require expert judgement: the user controls the compound and disease (or a custom target list), the choice of experimental structure among ranked candidates (including wild-type versus mutant forms), the binding-site and search-space definition, the covalent or non-covalent mode, and the search thoroughness. Automation is thus applied to the error-prone plumbing rather than to the analysis itself. This division is particularly important for structure selection, which carries limitations intrinsic to any metadata-driven choice. Because candidate structures are ranked from deposited metadata (experimental method, resolution, coverage and bound ligand) rather than from a conformational analysis, the top-ranked structure is not guaranteed to represent the biologically active conformation—a target may have been crystallised in an inactive, apo or alternative state, which should be verified before production docking. Domain coverage, likewise, is a global proxy for completeness and does not guarantee that every binding-site residue is resolved, since deposited structures frequently contain disordered or missing loops that the receptor-preparation step surfaces but cannot reconstruct de novo. Finally, where no experimental structure exists and an AlphaFold model is used, the prediction—reliable for well-folded domains but less certain in flexible or low-confidence regions, and lacking any experimental definition of the binding pocket in the absence of a co-crystallised ligand—warrants additional scrutiny and cross-checking against related experimental structures where possible before docking. In every case, DDS supports this expert review by presenting the recommended structure alongside its ranked alternatives, any mutant forms, and full metadata, so that the final choice rests with the user rather than with the automation.

Because DDS draws on live, independently maintained public databases whose contents are versioned and updated at provider-defined intervals, the output of an analysis reflects the state of those resources at the time of query: the pipeline is fully deterministic and reproducible for a given database state, but analyses separated in time may differ if an underlying resource has been updated, and results are therefore best interpreted with reference to their date of access.

## 3. Materials and Methods

This section describes the design of the DDS 2.0 network-pharmacology module in detail: the overall architecture, each analysis stage and the method, algorithm and data source used to implement it.

### 3.1. Overall Architecture and Design

DDS is a cross-platform desktop application (Microsoft Windows and Apple macOS) composed of two layers. The user interface is a web-technology front end (React 18.3.1, Python 3.9.6) that presents a guided, step-based workflow mirroring the layout of the DDS docking module, so that users familiar with one module can immediately use the other. Compute is handled by a local Python engine exposed as a lightweight web service (FastAPI/uvicorn); the same engine hosts the docking functionality of the previous DDS release, which is what makes the network-pharmacology-to-docking integration native rather than an external bridge. Long-running analyses execute as background jobs, so the interface remains responsive, and results persist while the user moves between steps and modules. Whereas the docking engine runs entirely offline, the network-pharmacology module is online by design: it queries public, programmatically accessible biological databases in real time. All queried resources are free and require no registration key, so the module works out of the box. Network operations use the NetworkX library [29]; interactive networks are rendered with Cytoscape.js 3.34.2 [30]; charts and figures are generated natively and exported as high-resolution PNG (transparent, white or dark background) or vector SVG; and tabular data and reports are packaged for download.

The workflow is organised as a sequence of steps, each feeding the next: (1) disease targets, (2) compound targets, (3) network (shared targets + PPI), (4) hub genes, (5) enrichment, and (6) structures for docking.

### 3.2. Disease-Associated Target Retrieval

Disease-associated genes are retrieved from the Open Targets Platform through its GraphQL API [10], which returns, for a queried disease, its associated targets together with an evidence-based association score (0–1) aggregating genetic, genomic, transcriptomic, pathway and literature evidence. Results are paginated exhaustively so that the full associated-gene set is obtained rather than a truncated top slice. Optionally, curated gene–disease associations from CTD [12] can be included. Retrieved genes are ranked by association score and can be filtered by a user-defined minimum-score cut-off. Importantly, users are not restricted to the built-in sources: a custom gene list from any external resource (e.g., GeneCards, OMIM, DisGeNET or a manually curated set) can be pasted or uploaded and is unioned with the database results, allowing the target library to be broadened as the study requires.

To ensure robustness, all external resources are queried with bounded request timeouts and within exception-handling wrappers, so that an unavailable, slow or malformed response from any single database neither stalls nor aborts an analysis: the affected source returns no contribution and the condition is surfaced to the user.

### 3.3. Compound–Target Prediction and Library Assembly

Compound targets are assembled from two complementary, key-free sources. Curated drug–gene interactions are obtained from DGIdb via its GraphQL API [13], and experimentally measured bioactivities are obtained from ChEMBL via its web-service API [14]; ChEMBL target resolution is parallelised to keep query times short. Each predicted target is assigned a normalised confidence score (0–1) reflecting the strength of the supporting evidence, and targets are ranked by confidence with a user-adjustable cut-off. As with disease targets, users may broaden the compound–target library by pasting or uploading externally predicted targets, for example from SwissTargetPrediction [15] or TCMSP [16], which are merged with the database-derived set. The compound can be specified by name or by SMILES string.

Because disease- and compound–target retrieval each draw on more than one independent source (Open Targets and CTD; ChEMBL and DGIdb), the workflow degrades gracefully when a source is temporarily unavailable, and structure retrieval falls back to the AlphaFold model where no experimental structure exists. Inconsistent gene identifiers across sources are reconciled by normalising symbols—including legacy and alias names—to their official HGNC symbols before intersection, so that cross-source matches are preserved.

### 3.4. Gene-Symbol Normalisation

Because different databases report gene identifiers inconsistently, all retrieved genes are normalised to official HGNC gene symbols using an internal alias table (for example, HER2 to ERBB2 and COX2 to PTGS2). Normalisation is applied before any set operation, so that combined libraries are clean, de-duplicated lists and cross-source intersections never miss a target owing to a synonym mismatch.

### 3.5. Shared-Target Identification

The shared targets—the intersection of the compound–target set and the disease-gene set—are computed and presented as an auto-labelled, exportable Venn diagram reporting the size of each region. These shared targets constitute the candidate therapeutic targets that are carried forward into the network analysis.

### 3.6. Protein–Protein Interaction (PPI) Network Construction

The shared-target set is submitted to the STRING database API [17] to retrieve experimentally supported and predicted protein–protein interactions at a user-selectable confidence threshold (default 0.4). The resulting interactions are assembled into a graph with NetworkX [29] and rendered as an interactive, concentric, degree-ranked network using Cytoscape.js [30], with node colour and size scaled to connectivity and an accompanying colour-scale legend.

### 3.7. Hub-Gene Identification

Hub genes are the most topologically central nodes of the PPI network and represent the most probable key regulators for downstream investigation. Centrality is computed with NetworkX [29]. The default metric is Maximal Clique Centrality (MCC), the method popularised by the cytoHubba Cytoscape plug-in [19], computed as the sum over all maximal cliques containing a node of (|C| − 1)!, where |C| is the clique size; maximal cliques are enumerated using the Bron–Kerbosch procedure. Degree, betweenness and closeness centrality are also provided and can be selected by the user. The top-ranked nodes are reported both on the network and as a ranked cytoHubba-style concentric figure in which the most central hub sits at the centre and node colour and size scale with the centrality score.

### 3.8. Functional Enrichment

The shared-target set is submitted to Enrichr [22] for over-representation analysis against Gene Ontology [20] (Biological Process, Molecular Function and Cellular Component) and KEGG pathways [21]. For each enriched term, DDS records the *p*-value, the Benjamini–Hochberg adjusted *p*-value (FDR), the Enrichr combined score, and the member genes. Enrichment results are presented as GO dot plots (term versus enrichment strength, with dot size scaled to the number of member genes and colour to −log10 FDR), a KEGG hub-gene-to-pathway Sankey diagram combined with a pathway lollipop/bubble plot, and a concentric compound–target–pathway network.

### 3.9. Multi-Compound and Multi-Disease Modes

Two batch modes extend the single-compound, single-disease workflow, with a deliberate semantic distinction. In multi-compound mode, several compounds are analysed together, and their targets are pooled as a union, with each target annotated by how many compounds engage it, so that broadly engaged targets rank higher; this is appropriate for multi-ingredient formulations or drug combinations. In multi-disease mode, several diseases are analysed together, and DDS takes the intersection of their gene sets—the shared, potentially comorbid-mechanism targets—which then proceed through the standard workflow. In both modes, the downstream analysis (shared targets, PPI, hub genes, enrichment) is unchanged; only the assembly of the target sets differs.

### 3.10. Covalent-Targetable Annotation

To connect network pharmacology with the covalent-docking capability of DDS, every target is cross-referenced against a curated reference of covalently ligandable proteins (proteins bearing targetable cysteine, catalytic serine/cysteine or other reactive nucleophilic residues) and flagged accordingly. Both the disease target and compound–target libraries can be filtered to covalent-targetable proteins only, allowing a covalent-focused network-pharmacology analysis that feeds naturally into the DDS covalent-docking protocols.

### 3.11. Selection of Mutant Target Structures

Because disease-relevant activity is often tied to specific mutant forms of a target—resistance-conferring substitutions in particular—DDS 2.0 makes the mutational status of each candidate structure explicit during hub-gene-to-structure mapping rather than restricting the workflow to the wild-type protein. For each prioritised hub gene, DDS resolves the gene to its reviewed human UniProt accession and retrieves the experimental structures cross-referenced for that accession in the Protein Data Bank (PDB). Each candidate is then annotated through a single RCSB PDB GraphQL query that returns, for the polymer entity matching the target’s UniProt accession, the number of engineered mutations (rcsb_mutation_count) and the corresponding amino-acid substitution(s) (pdbx_mutation); restricting this to the matching entity ensures that mutations carried by partner chains are not misattributed to the target. All candidates carrying one or more mutations are additionally collected into a dedicated list, each labelled with its explicit substitution (e.g., a point mutation such as EGFR T790M), and presented alongside the top-ranked (typically wild-type) structure. Importantly, mutant structures are identified from the annotations of the deposited experimental structures themselves—i.e., structures in which the variant has actually been resolved—rather than being imported from a separate clinical- or somatic-variant database, guaranteeing that every mutant corresponds to a real three-dimensional model. All data sources (UniProt, RCSB PDB, AlphaFold DB) are public and accessed without an API key.

### 3.12. Streamlined Structure Selection and Docking Integration

The distinguishing feature of DDS 2.0 is the integrated hand-off from hub genes to structure-based docking. For each top hub gene, DDS resolves a representative experimental structure through the following pipeline: the gene symbol is mapped to its reviewed human UniProt accession [31]; the cross-referenced PDB entries [23] are retrieved together with their experimental method, resolution, chain coverage and bound ligands (the latter via the RCSB PDB data API). Each candidate structure i is then ranked for docking suitability by a composite score, S_i_ = M_i_ + R_i_ + C_i_ + L_i_, that sums four weighted terms. The method term M rewards the experimental technique (40 for X-ray crystallography, 32 for cryo-electron microscopy, 10 for NMR and 6 for any other or undetermined method). The resolution term applies to resolution-defined methods as R = max(0, 30 − 12(d − 1.5)), where d is the resolution in Å, giving ≈30 points at 1.5 Å and decreasing linearly to 0 at ≥4.0 Å (R = 0 for NMR). The coverage term C = min(20, 20f) favours broader coverage of the mapped domain, f ∈ [0, 1] being the fraction of the UniProt sequence spanned by the structure. The ligand term L rewards—most importantly—the presence of a bound drug-like ligand, since a co-crystallised ligand both marks the true binding pocket and provides a natural centre for the docking search box: after excluding common crystallisation additives, ions and buffers, L = 25 for a ligand of molecular weight 150–700 Da, 14 for 700–1200 Da, 6 for 90–150 Da and 3 otherwise, with 8 assigned when a ligand is present but its molecular weight is undetermined and 0 when none qualifies. The top-ranked structure (maximal S_i_) is recommended and shown with its metadata and alternatives; when no experimental structure is available for a target, the corresponding AlphaFold [32] predicted model is offered as a fallback and is clearly labelled as such. These metadata-driven criteria resolve three further cases explicitly. Ties in the composite score are broken deterministically—in favour of, in order, the experimental method (X-ray > cryo-EM > NMR > other), then finer resolution, then greater domain coverage—so that selection is reproducible across runs. For entries containing several chains, each candidate is scored by the residue span of the target’s own UniProt sequence that it covers, independently of the number of chains or of unrelated partner chains present, so that homo- and hetero-multimeric structures are ranked by how completely they represent the target itself. Finally, because this stage ranks entries from their deposited metadata rather than from their atomic coordinates, resolution and coverage serve here as proxies for structural completeness; the explicit treatment of missing residues, alternate conformations and protonation is performed at the subsequent receptor-preparation step that precedes docking (described in the companion docking article).

With a single action, the selected structure is streamed into the DDS docking module: the receptor is fetched and prepared automatically, and the docking search box is pre-centred on the co-crystallised ligand, so the structure arrives docking-ready in the workspace shared with the covalent and non-covalent docking and virtual-screening protocols of the previous DDS release. This removes the manual literature search, structure selection, preparation and box-configuration steps that normally separate network pharmacology from docking. All structure data derive from public, key-free resources (UniProt, RCSB PDB and AlphaFold DB). Molecular docking itself is performed by the DDS engine using AutoDock Vina 1.2.0 [33].

### 3.13. Visualisation, Reporting and Export

All networks and figures are interactive and exportable. Networks (PPI, compound–target–pathway, hub-gene) and figures (Venn diagrams, GO dot plot, KEGG Sankey/bubble figure) can be exported as high-resolution PNG (transparent, white or dark background) or vector SVG for direct use in publications. A one-click report export produces (i) a full PDF report containing a summary, methods and protocol description, the disease- and compound–target tables, the shared-target set, the hub-gene ranking, the enrichment tables and references; and (ii) a compressed archive of comma-separated-value (CSV) files containing every analysis table (target libraries, intersections, PPI nodes and edges, hub genes and enrichment results) with descriptive file names, ready for spreadsheet analysis or archival as supplementary data.

### 3.14. Computational Resources, Reproducibility and Efficiency

All analyses were performed on a standard consumer laptop with no dedicated GPU or high-performance computing resources, and it operates within modest memory (a minimum of approximately 4 GB of RAM). The compute engine is implemented in Python (3.9+) and builds on established libraries. The installer bundles a self-contained Python runtime together with all required dependencies, so no separate Python installation or manual dependency management is required of the end user; for development, all dependencies are version-pinned in a requirements file in the source repository.

The workflow is CPU-light and memory-modest, so local computation is effectively instantaneous; a complete analysis, from compound and disease input to ranked hub genes, enriched pathways and docking-ready structures, typically completes within seconds to a few minutes, governed mainly by the response latency of the external services queried (STRING, Enrichr, UniProt, RCSB PDB and AlphaFold) rather than by on-device computation. Independent external queries are issued concurrently where possible, and each analysis runs as a background job, so that database latency is overlapped rather than accumulated.

As a representative example, the complete curcumin–triple-negative breast cancer benchmark analysis executed in approximately 30 s end-to-end on a standard consumer laptop, comprising disease- and compound–target retrieval with gene-symbol normalisation (≈6 s), STRING PPI network construction (≈4 s), hub-gene ranking by cytoHubba MCC (<1 s), GO/KEGG enrichment (≈5 s), and representative-structure selection for the ten hub genes (≈15 s); these times are dominated by external-service response latency and vary with network conditions, while on-device computation is effectively instantaneous.

The pipeline is fully streamlined and deterministic. Whereas a conventional analysis requires manual handling of data across several disconnected tools—target prediction, disease-gene retrieval, set intersection, STRING, Cytoscape/cytoHubba, an enrichment server and a separate structure-preparation step—DDS consolidates these into one guided interface with no intermediate file handling: the user supplies only the compound and disease, and the prioritised targets flow into enrichment and the docking-ready workspace. Analyses are reproducible for a given database state. A given input reproduces its entire output identically on re-execution and on other machines, and because every step is graphical rather than script-based, the platform is accessible to non-expert users. All external databases queried for the reported analyses—Open Targets, CTD, DGIdb, ChEMBL, STRING, Enrichr, UniProt, the RCSB Protein Data Bank and the AlphaFold Protein Structure Database—were accessed in June 2026; the reported results therefore correspond to the state of these resources at that date and should be interpreted accordingly.

## 4. Conclusions

Network pharmacology is now central to drug discovery, yet in practice it remains fragmented—a chain of separate databases and single-purpose tools that rarely connects to structure-based follow-up. DDS 2.0 removes this barrier by delivering the entire network-pharmacology workflow—disease- and compound–target assembly, gene-symbol normalisation, shared-target identification, PPI network construction, hub-gene ranking and GO/KEGG enrichment, with multi-compound and multi-disease modes and covalent-targetable annotation—within a single guided application, and—to our knowledge not offered by existing platforms—by streaming the prioritised hub genes as selected, docking-ready structures into the covalent- and non-covalent docking and virtual-screening engine of the previous DDS release. It thereby closes, in one user-installable platform, the loop from systems-level network analysis to covalent-aware structure-based design, with each analysis reproducible for a given database state and its results exportable as publication-ready figures, a report and data.

Future development will extend this integration further: incorporating additional biological databases and transcriptomic (expression) data to enrich target identification, adding interpretable machine learning models for target and activity prediction, maintaining alignment with successive AlphaFold updates, and providing an integrated hand-off to molecular-dynamics workflows for post-docking refinement—broadening DDS from an integrated discovery platform toward an end-to-end, systems-to-simulation environment.

## Figures and Tables

**Table 1:** Target retrieval and shared-target summary for the curcumin–TNBC benchmark.

Quantity	Value
TNBC-associated genes (Open Targets)	2057
Curcumin targets (DGIdb + ChEMBL)	117
Curcumin targets that are covalent-targetable	12
Shared targets (curcumin ∩ TNBC)	40
Shared targets that are covalent-targetable	6
PPI network nodes	40
PPI network edges	277

**Table 2:** Top 10 hub genes for the curcumin–TNBC benchmark, ranked by Maximal Clique Centrality (MCC, cytoHubba); the degree of each hub in the 40-node shared-target PPI network is also shown. Full centrality tables for all metrics are provided in the Supplementary Material File S3.

Rank	Gene	Degree
1	STAT3	31
2	TNF	32
3	AKT1	32
4	BCL2	29
5	GSK3B	21
6	EGFR	31
7	PPARG	18
8	PTGS2	24
9	AR	18
10	NFE2L2	15

**Table 3:** Representative enriched terms for the curcumin–TNBC benchmark. Complete statistics are provided in the Supplementary Material File S3.

Category	Representative Enriched Terms
GO Biological Process	positive/negative regulation of transcription (RNA-polymerase-II and DNA-templated); regulation of cell growth; regulation of apoptotic process; negative regulation of programmed cell death; regulation of inflammatory response; protein phosphorylation
KEGG pathways	Pathways in cancer; Prostate cancer; Pancreatic cancer; Chemical carcinogenesis; PD-L1 expression and PD-1 checkpoint pathway in cancer; cancer-associated viral infections (hepatitis B/C, human papillomavirus)

**Table 4:** Benchmark comparison of the DDS 2.0 network-pharmacology workflow against the published reference study [24] for curcumin in triple-negative breast cancer.

Parameter	Reference Study [25]	DDS 2.0 (This Work)
Disease studied	Triple-negative breast cancer (TNBC)	Triple-negative breast cancer (TNBC)
Disease targets (source)	2060 (OMIM, TTD, DisGeNET)	2057 (reference target set, supplied via DDS upload)
Curcumin targets (source)	118 (SwissTargetPrediction 110; ETCM 8)	117 (reference target set, supplied via DDS upload)
Shared targets (intersection)	40	40
PPI network	STRING	STRING (conf. 0.4); 40 nodes, 277 edges
Hub-gene ranking method	Cytoscape cytoHubba, MCC	cytoHubba MCC (degree/betweenness/closeness also available)
Top 10 hub genes	STAT3, AKT1, TNF, PTGS2, MMP9, EGFR, PPARG, NFE2L2, EP300, GSK3B	STAT3, TNF, AKT1, BCL2, GSK3B, EGFR, PPARG, PTGS2, AR, NFE2L2
Reference hubs recovered by DDS	(reference set)	8/10 in DDS top 10 (STAT3, AKT1, TNF, PTGS2, EGFR, PPARG, NFE2L2, GSK3B); the 2 misses (MMP9, EP300) present in the network, just outside the top 10
Key enriched pathways	PI3K–Akt; EGFR TKI resistance; JAK–STAT; PD-L1/PD-1 checkpoint; microRNAs in cancer; Chemical carcinogenesis–receptor activation	Pathways in cancer; Prostate/Pancreatic cancer; Chemical carcinogenesis; PD-L1/PD-1 checkpoint pathway in cancer; cancer-associated viral infections

**Table 5:** Representative selected structures for benchmark hub genes. Full structure tables are provided in the Supplementary Material File S3.

Hub Gene	UniProt	PDB	Method	Res. (Å)	Bound Ligand
STAT3	P40763	6NJS	X-ray	2.7	SD36 (drug-like inhibitor)
TNF	P01375	9OJO	X-ray	1.36	small-molecule inhibitor (A1CB1)
AKT1	P31749	4GV1	X-ray	1.49	AZD5363/capivasertib (0XZ)

## Data Availability

DDS is a cross-platform desktop application. Full analysis tables, data and the generated report for the benchmark case study are provided as Supplementary Material Files S1–S3. DDS 2.0 is freely and publicly accessible via (http://soliman.ukzn.ac.za/DDS2.aspx, http://soliman.ukzn.ac.za/DrugDesignStudio.aspx, https://drive.google.com/drive/u/1/folders/157NyhAYp0nTZmKdeKUQYjhXeu3fNjVzK (accessed on 31 August 2026)). Comprehensive user documentation is built directly into DDS and can be accessed at any time from the Documentation panel. The original contributions presented in this study are included in the article/Supplementary Materials. Further inquiries can be directed to the corresponding author.

## Supplementary Materials

The following supporting information can be downloaded at: https://www.mdpi.com/article/10.3390/ijms27177874/s1. References [34,35,36,37,38] are cited in the supplementary materials.
