# Supplementary material for: Drug Design Studio (DDS) 2.0: A Unified Platform for Network Pharmacology Integrated with Docking and Virtual Screening Workflow for Covalent/Non-Covalent Binders
Source: Int J Mol Sci. 2026 Sep 3;27(17):7874. doi: 10.3390/ijms27177874 (PMC13566856; doi:10.3390/ijms27177874)
Supplement: Supplementary file 1 [file ijms-27-07874-s001.zip › File S2.pdf]

Network Pharmacology Report

CURCUMIN × triple-negative breast carcinoma · generated 2026/07/26, 13:25:06 · Drug Design Studio (DDS)

Note: This report is intended as guidance for your write-up. Please do not copy it verbatim — rephrase the findings in your own words.

1. Summary

This report describes a network-pharmacology analysis linking **CURCUMIN** to **triple-negative breast carcinoma**. Compound targets and disease-associated genes were assembled, their shared targets identified, a protein–protein interaction (PPI) network constructed, hub genes ranked, and functional enrichment computed. The intersection yielded **84** shared targets, a PPI network of **81** nodes and **688** edges, and the top hub genes below.

2. Methods & protocol

Compound targets. Targets were retrieved for the compound. Targets came from DGIdb (curated drug–gene interactions) and ChEMBL (measured bioactivity), mapped to a 0–1 confidence and de-duplicated to official gene symbols.

Disease targets. Disease-associated genes were retrieved and ranked by association score. Sources: Open Targets (evidence-scored) and CTD, unioned with any user-supplied list.

Network & hubs. Shared targets (compound ∩ disease) were submitted to STRING (confidence ≥ 0.4) to build the PPI network; hub genes were ranked by degree centrality (cytoHubba-style) using NetworkX.

Enrichment. Gene-set over-representation was computed with Enrichr for: GO — Biological process, KEGG pathways.

All computations are illustrative of the software's output; results reflect the state of the underlying public databases at 2026/07/26, 13:25:06.

3. Disease targets

2490 disease-associated genes, of which **110** are covalent-targetable. Top by association score:

#	Gene	Association score	Covalent
1	TACSTD2	0.551	

#	Gene	Association score	Covalent
2	TOP1	0.546	
3	FTO	0.488	
4	BRCA1	0.477	
5	PDCD1	0.424	
6	TUBB2B	0.424	
7	TUBB3	0.424	
8	TUBB2A	0.423	
9	TUBA1C	0.422	
10	CD274	0.421	
11	TUBA1A	0.421	
12	TUBA1B	0.421	
13	TUBA3C	0.421	
14	TUBA3E	0.421	
15	TUBA4A	0.421	
16	TUBB	0.421	
17	TUBB1	0.421	
18	TUBB4A	0.421	
19	TUBB4B	0.421	
20	TUBB6	0.421	
21	TUBB8	0.421	
22	TCF7L2	0.419	
23	TOX3	0.415	
24	TYMS	0.414	
25	TERT	0.412	

#	Gene	Association score	Covalent
26	POLD2	0.405	
27	RRM2	0.405	
28	POLD1	0.404	
29	RRM1	0.404	
30	POLA1	0.403	

4. Compound targets

134 compound targets, **17** covalent-targetable. Top by confidence:

#	Gene	Confidence	Source	Covalent
1	APP	0.95	chembl	
2	DFFB	0.95	dgidb	
3	DYRK2	0.77	chembl	
4	GLO1	0.72	chembl	⚡
5	FOS	0.69	chembl	
6	JUN	0.69	chembl	
7	F3	0.65	chembl	
8	HSD17B3	0.65	dgidb	
9	THBD	0.58	dgidb	
10	EP300	0.57	dgidb	
11	PTGES	0.57	dgidb	
12	STAT3	0.55	dgidb	⚡
13	DNMT3B	0.52	dgidb	
14	TRPV4	0.52	dgidb	

#	Gene	Confidence	Source	Covalent
15	CISD1	0.5	chembl	
16	MMP9	0.49	dgidb	
17	WT1	0.48	dgidb	
18	DYRK1B	0.47	chembl	
19	IAPP	0.46	dgidb	
20	UGT1A7	0.46	dgidb	
21	GSTM1	0.45	dgidb	⚡
22	ANPEP	0.44	dgidb	
23	CAMK2A	0.44	dgidb	
24	CCN2	0.44	dgidb	
25	SNCA	0.44	chembl	
26	DDIT3	0.43	dgidb	
27	PTGS1	0.43	dgidb	⚡
28	SMAD2	0.43	dgidb	
29	BDNF	0.42	dgidb	
30	CA6	0.42	dgidb	

5. Shared targets (compound ∩ disease)

84 targets are shared between the compound and disease sets — the candidate therapeutic targets carried into the network analysis.

APP, GLO1, FOS, JUN, F3, THBD, EP300, STAT3, DNMT3B, CISD1, MMP9, WT1, GSTM1, CAMK2A, CCN2, DDIT3, PTGS1, SMAD2, BDNF, DYRK1A, GSTP1, PTGS2, ABCB1, CA12, DNMT1, CYP3A4, EGFR, ERBB2, ERBB3, ERBB4, GLS, GMNN, HPGD, MAPT, NFE2L2, NOS2, PTGER2, SMAD3, TOP1, TUBA1A, TUBA1B, TUBA1C, TUBA3C, TUBA3E, TUBA4A, TUBB, TUBB1, TUBB2A, TUBB2B, TUBB3, TUBB4A, TUBB4B, TUBB6, TUBB8, USP1, USP2, VDR, HDAC1, HDAC10, HDAC11, HDAC2, HDAC3, HDAC4, HDAC5, HDAC6, HDAC7, HDAC8, HDAC9, ABCG2, CA9, KMT2A, BRCA1, CLK1, HIF1A, ATP2A2, NFKB1, NFKB2, RELA, TLR9, ALDH1A1, KDM1A, MAPK1, PRKCE, PSMB5

6. Hub genes (degree centrality)

Hub genes are the most topologically central nodes in the PPI network — the most likely key regulators for further study.

Rank	Gene	Centrality score	Degree	Hub
1	EGFR	45	45	★
2	HIF1A	44	44	★
3	STAT3	41	41	★
4	JUN	40	40	★
5	EP300	38	38	★
6	NFKB1	37	37	★
7	HDAC6	32	32	★
8	PTGS2	32	32	★
9	HDAC1	31	31	★
10	DNMT1	29	29	★
11	ERBB2	29	29	
12	FOS	29	29	
13	SMAD3	29	29	
14	BRCA1	27	27	
15	HDAC2	27	27	

7. Functional enrichment

GO — Biological process

Term	p-value	Adj. p	Combined	Genes
positive regulation of transcription, DNA-templated (GO:0045893)	2.2e-15	1.4e-12	289	29
positive regulation of transcription by RNA polymerase II (GO:0045944)	2.4e-15	1.4e-12	326	26

Term	p-value	Adj. p	Combined	Genes
protein deacetylation (GO:0006476)	2.8e-15	1.4e-12	2573	10
histone deacetylation (GO:0016575)	4.5e-15	1.7e-12	2399	10
regulation of gene expression (GO:0010468)	1.4e-13	4.3e-11	238	26
positive regulation of nucleic acid-templated transcription (GO:1903508)	2.8e-13	7.3e-11	334	19
negative regulation of transcription by RNA polymerase II (GO:0000122)	5.2e-13	1.0e-10	274	21
negative regulation of transcription, DNA-templated (GO:0045892)	5.3e-13	1.0e-10	232	24
histone H4 deacetylation (GO:0070933)	9.5e-13	1.6e-10	10601	6
protein deacylation (GO:0035601)	4.1e-12	6.5e-10	2963	7
cellular response to reactive oxygen species (GO:0034614)	5.2e-12	7.3e-10	1147	9
negative regulation of nucleic acid-templated transcription (GO:1903507)	7.5e-12	9.7e-10	283	17

KEGG pathways

Term	p-value	Adj. p	Combined	Genes
Gap junction	3.4e-24	6.7e-22	3832	17
Alzheimer disease	1.1e-23	9.4e-22	1274	25
Pathways of neurodegeneration	1.4e-23	9.4e-22	1083	27
Pathogenic Escherichia coli infection	1.7e-22	8.4e-21	1747	20
Salmonella infection	6.9e-22	2.7e-20	1402	21
Parkinson disease	2.0e-20	6.4e-19	1219	20
Viral carcinogenesis	3.2e-19	9.1e-18	1239	18
Huntington disease	1.2e-18	2.9e-17	885	20
Pathways in cancer	2.0e-17	4.3e-16	554	23
MicroRNAs in cancer	3.2e-17	6.2e-16	749	19
Amyotrophic lateral sclerosis	3.5e-17	6.3e-16	674	20

Term	p-value	Adj. p	Combined	Genes
Prion disease	6.5e-17	1.1e-15	784	18

References

1. Hopkins AL. Network pharmacology: the next paradigm in drug discovery. *Nat Chem Biol* 2008;4:682–90.
2. Ochoa D, et al. The Open Targets Platform. *Nucleic Acids Res* 2023;51:D1353–9.
3. Davis AP, et al. Comparative Toxicogenomics Database (CTD). *Nucleic Acids Res* 2023;51:D1257–62.
4. Freshour SL, et al. Drug–Gene Interaction Database (DGIdb 4.0). *Nucleic Acids Res* 2021;49:D1144–51.
5. Zdrazil B, et al. The ChEMBL Database in 2023. *Nucleic Acids Res* 2024;52:D1180–92.
6. Szklarczyk D, et al. STRING v12. *Nucleic Acids Res* 2023;51:D638–46.
7. Chin CH, et al. cytoHubba: identifying hub objects and sub-networks. *BMC Syst Biol* 2014;8(S4):S11.
8. Hagberg AA, et al. Exploring network structure with NetworkX. *Proc. SciPy* 2008:11–15.
9. Kuleshov MV, et al. Enrichr. *Nucleic Acids Res* 2016;44:W90–7.
10. Soliman ME. Drug Design Suite (DDS) 2.0: A Unified Platform for Network Pharmacology Integrated with Docking and Virtual Screening Workflow for Covalent/Non-Covalent Binders. *(Under review)*. Molecular Bio-computation & Drug Design Laboratory, UKZN.
