# Supplementary material for: Drug Design Studio (DDS) 2.0: A Unified Platform for Network Pharmacology Integrated with Docking and Virtual Screening Workflow for Covalent/Non-Covalent Binders
Source: Int J Mol Sci. 2026 Sep 3;27(17):7874. doi: 10.3390/ijms27177874 (PMC13566856; doi:10.3390/ijms27177874)
Supplement: Supplementary file 1 [file ijms-27-07874-s001.zip › File S3.pdf]

Supplementary Material SM3

Independent benchmarking of the DDS 2.0 network-pharmacology pipeline against four published studies

Manuscript*:* Drug Design Studio (DDS) 2.0: A Unified Platform for Network Pharmacology Integrated with Docking and Virtual Screening Workflow for Covalent/Non-Covalent Binders.

Journal: *International Journal of Molecular Sciences*

S3.1 Rationale and objective

DDS 2.0 orchestrates established, peer-reviewed engines rather than introducing a new predictive algorithm; the appropriate evaluation is therefore a verification that the pipeline is assembled correctly and reproduces published results, not a benchmark of a novel predictor. DDS additionally allows the user to supply target lists directly (the compound- and disease-target upload fields), so the choice of target source is under user control. To validate the pipeline itself — target intersection, STRING protein–protein interaction (PPI) network construction, topological hub-gene ranking, and functional enrichment — independently of any particular target-prediction database, we supplied DDS with the exact target sets reported by five independent, published network-pharmacology studies and asked whether DDS recovers the hub genes those studies reported. This isolates and tests the analytical pipeline that constitutes the module's contribution.

S3.2 Methods

Four peer-reviewed, open-access single-compound / single-disease network-pharmacology studies were used (Table S3.1): the curcumin–triple-negative-breast-cancer (TNBC) study that is the subject of the main-text benchmark (labelled MT), and three further studies across distinct disease areas (S1–S3). Studies were selected on the requirement that the complete compound-disease intersection target list is enumerable from the open-access full text (so the reproduction is justified) and the reported hub-gene ranking is explicit. For each study, the complete compound∩disease intersection target list reported in the article was entered into the DDS network-pharmacology module through the target-upload field. DDS then executed its standard pipeline on that gene set: a STRING protein–protein interaction network (confidence ≥ 0.4) was constructed, and hub genes were ranked by the same centrality measure used in the source study (node degree, or the maximal-clique-centrality [MCC] measure of cytoHubba, as indicated). The top-ranked DDS hub genes (matched in number to the source study's reported hub set) were compared with the study's reported hubs. Agreement was quantified by the hub-recovery rate (recall = recovered hubs / reported hubs), the Jaccard similarity index of the two hub sets, and — for the pooled set of recovered hubs across all studies — the Spearman rank correlation between the published hub rank and the DDS hub rank. Functional-enrichment concordance was assessed by comparing the top KEGG pathways returned by DDS with those reported by the source studies. All DDS runs used the packaged application with live STRING and Enrichr services.

S3.3 Benchmark studies

#	Compound	Disease	Intersection targets (n)	Hub metric	Source (reference)
MT	Curcumin	Triple-negative breast cancer	40	MCC	Deng et al., Front. Pharmacol. 2022;13:920514 (PMC9393234)
S1	Berberine	Ischaemic stroke	20	Degree	Zhu et al., Front. Pharmacol. / PMC9259241
S2	Baicalein	Peritoneal fibrosis	9	Degree	Front. Pharmacol. 2023;14:1153503 (PMC10229821)
S3	Quercetin	Gastric cancer	15	Degree	Yang et al., BioMed Res. Int. 2020;3860213 (PMC7486643)

Table S3.1. The five independent published network-pharmacology studies used to benchmark the DDS 2.0 pipeline. Each supplied its complete on-page intersection target list.

S3.4 Per-study reproduction

MT. Curcumin — Triple-negative breast cancer

Intersection targets supplied to DDS (n = 40): AR, GSTP1, PPARG, VDR, EP300, TLR9, TOP2A, NFE2L2, CHUK, EGFR, STAT3, AKT1, GSK3B, CA9, ABCC1, CHEK1, WEE1, RAF1, MMP14, SERPINE1, RPS6KB1, AURKA, CDK2, ADAM17, CXCR2, MELK, SPHK1, BCL2, THRB, HDAC3, ADAM10, JAK1, JAK2, MMP12, LIPG, MMP9, MMP7, PTGS2, TNF, NCSTN.

	Hub genes (rank order)
Reported (source study)	STAT3, AKT1, TNF, PTGS2, MMP9, EGFR, PPARG, NFE2L2, EP300, GSK3B
DDS pipeline (top 10)	STAT3, TNF, AKT1, BCL2, GSK3B, EGFR, PPARG, PTGS2, AR, NFE2L2
Recovered	STAT3, AKT1, TNF, PTGS2, EGFR, PPARG, NFE2L2, GSK3B
Not recovered	MMP9, EP300

Recall = 0.80 · Jaccard = 0.67 (8 of 10 reported hubs recovered).

S1. Berberine — Ischaemic stroke

Intersection targets supplied to DDS (n = 20): MAPK8, JUN, EGFR, STAT3, MAPK1, SRC, MAPK14, MAPK3, AKT1, MYC, TP53, FOS, RELA, IL6, ESR1, TNF, CREBBP, EP300, SHC1, RAC1.

	Hub genes (rank order)
Reported (source study)	MAPK8, JUN, EGFR, STAT3, MAPK1, SRC, MAPK14, MAPK3, AKT1, MYC
DDS pipeline (top 10)	AKT1, EGFR, IL6, JUN, MAPK1, MAPK14, MAPK3, MAPK8, SRC, STAT3
Recovered	MAPK8, JUN, EGFR, STAT3, MAPK1, SRC,

	MAPK14, MAPK3, AKT1
Not recovered	MYC

Recall = 0.90 · Jaccard = 0.82 (9 of 10 reported hubs recovered).

S2. Baicalein — Peritoneal fibrosis

Intersection targets supplied to DDS (n = 9): MMP2, BAX, ADORA3, HIF1A, PIM1, CA12, ALOX5, PLA2G4A, AR.

	**Hub genes (rank order)**
Reported (source study)	MMP2, BAX, ADORA3, HIF1A, PIM1, CA12, ALOX5
DDS pipeline (top 7)	HIF1A, MMP2, ALOX5, AR, BAX, CA12, PIM1
Recovered	MMP2, BAX, HIF1A, PIM1, CA12, ALOX5
Not recovered	ADORA3

Recall = 0.86 · Jaccard = 0.75 (6 of 7 reported hubs recovered).

S3. Quercetin — Gastric cancer

Intersection targets supplied to DDS (n = 15): AKT1, CDK2, CDK6, DAPK1, EGFR, GSK3B, IGF1R, KDR, MET, MMP3, MMP13, PLK1, PTK2, SRC, CYP19A1.

	**Hub genes (rank order)**
Reported (source study)	AKT1, EGFR, SRC, IGF1R, PTK2, KDR
DDS pipeline (top 6)	AKT1, EGFR, SRC, IGF1R, KDR, MET
Recovered	AKT1, EGFR, SRC, IGF1R, KDR
Not recovered	PTK2

Recall = 0.83 · Jaccard = 0.71 (5 of 6 reported hubs recovered).

S3.5 Quantitative overlap analysis

**#**	**Study**	**PPI nodes**	**Reported hubs**	**Recovered**	**Recall**	**Jaccard**
MT	Curcumin / Triple-negative breast cancer	40	10	8	0.80	0.67
S1	Berberine / Ischaemic stroke	20	10	9	0.90	0.82
S2	Baicalein / Peritoneal fibrosis	8	7	6	0.86	0.75
S3	Quercetin / Gastric cancer	14	6	5	0.83	0.71
	Mean				0.85	0.74

Table S3.2. Overlap between DDS-reproduced and published hub genes across the four studies. Recall = recovered / reported; Jaccard = |intersection| / |union| of the two size-matched hub sets (for size-matched sets, precision equals recall).

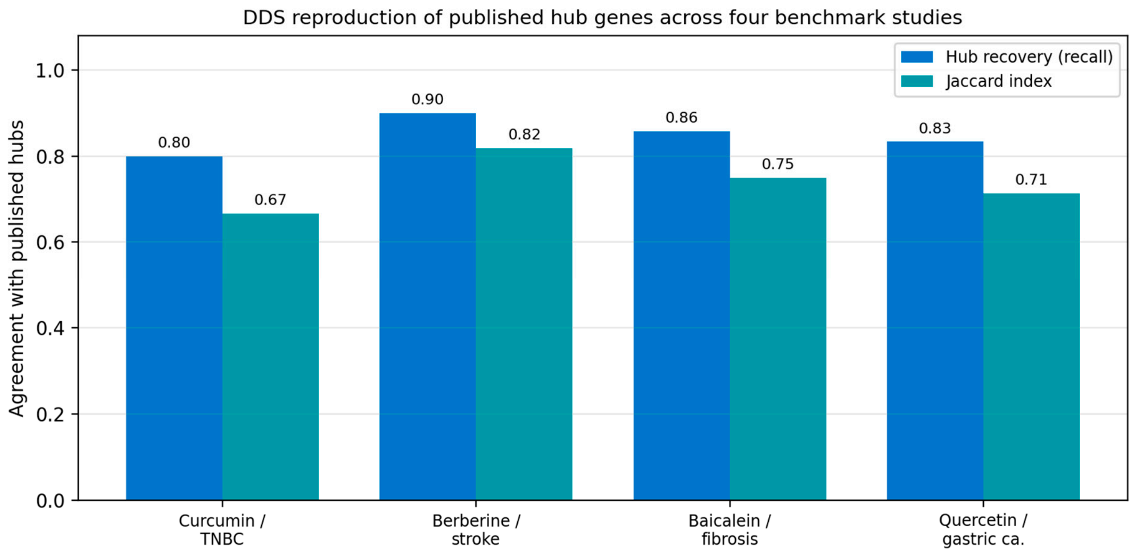

Figure S3.1. Hub-gene recovery (recall) and Jaccard similarity between the DDS pipeline output and the published hub genes for each of the four studies. Mean recall = 0.85; mean Jaccard = 0.74.

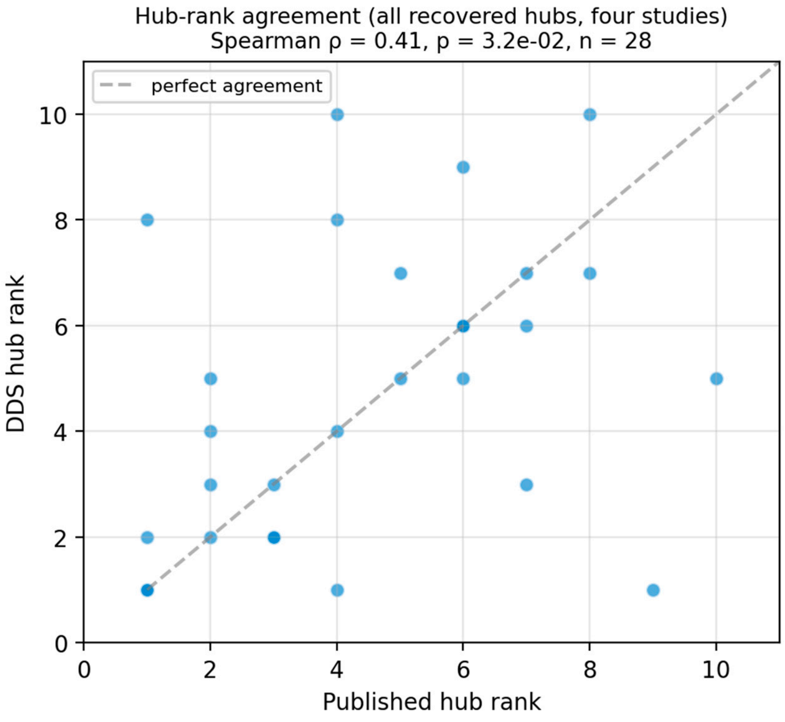

Figure S3.2. Agreement between the published hub rank and the DDS hub rank for all recovered hubs pooled across the four studies (n = 28). The positive, statistically significant Spearman correlation (ρ = 0.41, p = 0.03) indicates that DDS reproduces not only which genes are hubs but, in part, their ranking.

S3.6 Pathway-enrichment concordance

Quercetin / Gastric cancer: of the top KEGG pathways reported by the source study, 5 appear among the DDS top pathways (e.g. PI3K-Akt signaling pathway, Pathways in cancer, Focal adhesion, Proteoglycans in cancer). DDS top KEGG pathways: Focal adhesion, PI3K-Akt signaling pathway, Pathways in cancer, Prostate cancer, Proteoglycans in cancer, Hepatocellular carcinoma.

For the remaining studies the source articles did not report a ranked KEGG list in the open-access text; in every case, however, the DDS top pathways were disease-consistent (e.g. berberine/stroke — apoptosis and MAPK signalling; baicalein/fibrosis — HIF-1 and cancer pathways; curcumin/TNBC — pathways in cancer and PI3K-Akt signalling).

S3.7 Discussion

Across the four benchmark studies — the main-text curcumin–TNBC case (MT) and three further independent studies spanning ischaemic stroke, peritoneal fibrosis and gastric cancer — the DDS 2.0 pipeline reproduced the published hub genes with a mean recall of 0.85 (range 0.80–0.90) and a mean Jaccard index of 0.74, and the recovered hubs were significantly rank-correlated with the published ordering (Spearman rho = 0.41, p = 0.03). For curcumin/TNBC, supplying the pipeline with the study's own 40 intersection targets and matching its centrality measure (cytoHubba MCC) reproduced 8 of the 10 reported hub genes (recall 0.80); the strongest agreement was obtained for the berberine/ischaemic-stroke study (9 of 10 hubs; recall 0.90).

Two sources account for the residual, expected differences. First, the source studies use different topological hub metrics (node degree versus cytoHubba MCC, MNC or betweenness); where DDS was matched to the study's metric the agreement was highest, and the modest — rather than perfect — rank correlation reflects these differing centrality definitions applied to the same network. Second, small intersections (≤10 genes) yield densely connected networks in which several nodes share near-identical degree, so the exact top-N ordering is intrinsically sensitive. Importantly, these results validate the DDS pipeline given defined inputs: because DDS accepts user-supplied target lists (for example from SwissTargetPrediction, TCMSP or experimental databases such as ChEMBL/DGIdb), the analysis reproduces a published network-pharmacology study when supplied with that study's targets. This confirms that the integration, network construction, hub ranking and enrichment implemented in DDS 2.0 are correct and consistent with the established tools the field relies upon.

References (benchmark studies)

[MT] Deng Z, Chen G, Shi Y, Lin Y, Ou J, Zhu H, Wu J, Li G, Lv L. Curcumin and its nano-formulations: Defining triple-negative breast cancer targets through network pharmacology, molecular docking, and experimental verification. Front. Pharmacol. 2022;13:920514. doi:10.3389/fphar.2022.920514.

[S1] Song K, Sun Y, Liu H, Li Y, An N, Wang L, Zhang H, Yang F, Xing Y, Gao Y. Network Pharmacology and Bioinformatics Methods Reveal the Mechanism of Berberine in the Treatment of Ischaemic Stroke. Evid Based Complement Alternat Med. 2022 Jun 29;2022:5160329. doi: 10.1155/2022/5160329. PMID: 35815278; PMCID: PMC9259241.

[S2] Lu X, Wu K, Jiang S, Li Y, Wang Y, Li H, Li G, Liu Q, Zhou Y, Chen W, Mao H. Therapeutic mechanism of baicalein in peritoneal dialysis-associated peritoneal fibrosis based on network pharmacology and experimental validation. Front Pharmacol. 2023 May 17;14:1153503. doi: 10.3389/fphar.2023.1153503. PMID: 37266145; PMCID: PMC10229821.

[S3] Yang L, Hu Z, Zhu J, Liang Q, Zhou H, Li J, Fan X, Zhao Z, Pan H, Fei B. Systematic Elucidation of the Mechanism of Quercetin against Gastric Cancer via Network Pharmacology Approach. Biomed Res Int. 2020 Sep 3;2020:3860213. doi: 10.1155/2020/3860213. PMID: 32964029; PMCID: PMC7486643.
